# Impact of Tm^3+^ and Tb^3+^ Rare Earth Cations Substitution on the Structure and Magnetic Parameters of Co-Ni Nanospinel Ferrite

**DOI:** 10.3390/nano10122384

**Published:** 2020-11-29

**Authors:** Munirah A. Almessiere, Yassine Slimani, İsmail A. Auwal, Sagar E. Shirsath, Ayyar Manikandan, Abdulhadi Baykal, Bekir Özçelik, İsmail Ercan, Sergei V. Trukhanov, Denis A. Vinnik, Alex V. Trukhanov

**Affiliations:** 1 Department of Biophysics, Institute for Research and Medical Consultations (IRMC), Imam Abdulrahman Bin Faisal University, P.O. Box 1982, Dammam 31441, Saudi Arabia; yaslimani@iau.edu.sa (Y.S.); iercan@iau.edu.sa (İ.E.); 2 Department of Chemistry, Sule Lamido University, P.M.B 048 Kafin Hausa, Jigawa State, Nigeria; ismaila.auwal@slu.edu.ng; 3 School of Materials Science and Engineering, University of New South Wales, Sydney 2052, Australia; s.shirsath@unsw.edu.au; 4 Department of Chemistry, Bharath Institute of Higher Education and Research (BIHER), Bharat University, Chennai 600073, India; mkavath15@gmail.com; 5 Department of Nanomedicine Research, Institute for Research and Medical Consultations (IRMC), Imam Abdulrahman Bin Faisal University, P.O. Box 1982, Dammam 31441, Saudi Arabia; abaykal@iau.edu.sa; 6 Department of Physics, Faculty of Science, Çukurova University, Adana 01330, Turkey; ozcelik@cu.edu.tr; 7 Laboratory of Magnetic Films Physics, SSPA “Scientific and Practical Materials Research Centre of NAS of Belarus”, 220072 Minsk, Belarus; sv_truhanov@mail.ru; 8 Laboratory of Single Crystals Growth, Scientific and Educational Center “Nanotechnology”, South Ural State University, 454080 Chelyabinsk, Russia; denisvinnik@gmail.com; 9 Department of Electronic Materials Technology, Institute of New Materials and Nanotechnology, National University of Science and Technology MISiS, 119049 Moscow, Russia

**Keywords:** nanospinel ferrites, rare-earth elements, microstructure, morphology, magnetic features

## Abstract

Tm-Tb co-substituted Co-Ni nanospinel ferrites (NSFs) as (Co_0.5_Ni_0.5_) [Tm_x_Tb_x_Fe_2−2x_]O_4_ (x = 0.00–0.05) NSFs were attained via the ultrasound irradiation technique. The phase identification and morphologies of the NSFs were explored using X-rays diffraction (XRD), selected area electron diffraction (SAED), and transmission and scanning electronic microscopes (TEM and SEM). The magnetization measurements against the applied magnetic field (M-H) were made at 300 and 10 K with a vibrating sample magnetometer (VSM). The various prepared nanoparticles revealed a ferrimagnetic character at both 300 and 10 K. The saturation magnetization (M_s_), the remanence (M_r_), and magneton number ($n_{B}$) were found to decrease upon the Tb-Tm substitution effect. On the other hand, the coercivity (H_c_) was found to diminish with increasing x up to 0.03 and then begins to increase with further rising Tb-Tm content. The H_c_ values are in the range of 346.7–441.7 Oe at 300 K to 4044.4–5378.7 Oe at 10 K. The variations in magnetic parameters were described based on redistribution of cations, crystallites and/or grains size, canting effects, surface spins effects, super-exchange interaction strength, etc. The observed magnetic results indicated that the synthesized (Co_0.5_Ni_0.5_)[Tm_x_Tb_x_Fe_2−x_]O_4_ NSFs could be considered as promising candidates to be used for room temperature magnetic applications and magnetic recording media.

## 1. Introduction

The complex oxides of transition metals are attractive both from a practical application [1,2,3] and from the point of view of studying the fundamental physical phenomena [4,5,6]. Close attention to these compounds appeared due to the interconnection of structural, charge, and spin interactions [7]. Multiferroics are the most relevant for research in the physics and chemistry of condensed matter [8,9,10]. Ferrites stand out among the most promising multiferroics [11,12,13].

The broad field of science and technology of spinel ferrite materials has been established [14,15,16]. Certainly, magnetic nanocrystal ferrites have the potentials for use as raw material for various technological industries processes, such as in non-volatile memory devices, spintronics, and energy storage devices [17,18,19], and also in bio-implementations, such as medical imaging, nano-drug delivery, and sensing [20,21]. The structure of spinel cubic ferrite contains a divalent cation (often transition metals) in addition to ferric ion, represented as Me^2+^Fe_2_^3+^O_4_, where metals are bound by oxygen to form a face-centered cubic closed packing. Each of the cations resides either at tetrahedral *A* or an octahedral *B* lattice site [22]. Ferrites and derivatives are known for their broad ferromagnetic resonance linewidth, moreover, owing to their exceptionally high magnetic and dielectric loss, these crystals are regarded as crucial particularly in the area of electromagnetic interference and low observable technology (LOT) [23,24,25].

Typically, a candidate material for use as an electromagnetic resonance absorber should have (i) broad magnetic resonance bandwidth, large values of (ii) permeability, and (iii) permittivity [23,25]. NiCoFe_3_O_4_ nanomaterials are reported to have a low coercivity (1.20 Oe) and high values of saturation magnetic flux density (2.1 T) [26] and Curie temperature (~527 °C), high permeability and permittivity, and low value of magnetocrystalline anisotropy and appreciable resistivity (~10^5^–10^4^ ohm/cm). In addition, it is well known that both Co- and Ni are inverse spinels, that exhibit homogeneous distribution in the A and B lattice sites; thus, their magnetic features are attributed to antiferromagnetic coupling amongst the tetrahedral and octahedral sublattices [27,28]. Furthermore, nickel substituted cobalt ferrites demonstrate good chemical stability and thus found applications as a binder and as magnetic filler in electromagnetic shielding nanocomposites [29,30,31]. Moreover, it is generally anticipated that rare-earth substituted spinel ferrites, in many cases, would show a superior electric and magnetic character when compared with the pristine spinel counterpart [32].

The bottom-up chemical synthesis routes commonly employed to prepare magnetic nano ferrites include co-precipitation, thermal decomposition, microemulsion, hydrothermal, and polyol methods [33,34,35,36]. Although these techniques yield materials with a narrow size distribution, precise control of the structural morphology, surface area, and size/size distribution during the chemical reaction is challenging [37]. Additionally, large scale production of uniformly sized magnetic nanoparticles is crucial since many advanced technological applications are sensitive not only to nano-sized crystals but a material with crystals size consistency [38]. For instance, studies have shown that size/size distribution is a major factor that determines the mechanical and thermal stability of magnetic nanofluid that are used as heat transfer media [39]. Fortunately, a strict size control can be achieved through the sonochemical approach. This technique follows the acoustic cavitation phenomenon [37], where the reaction mixture is subjected to ultrasound treatment at high temperature and pressure. Consequently, the reaction is quenched by rapid cooling followed by the application of strong shock waves and microjets [37,40]. This process hinders secondary nucleation and offers excellent size/size distribution. In addition, the sonochemical method is inexpensive, environmentally benign, effective, and most importantly, produces magnetic nanocrystal with a better magnetic response.

Spinel ferrites substituted with rare-earth ions have been reported by numerous research groups. Biasi and co-workers investigated the magnetocrystalline anisotropy of NiCo_x_Fe_2−x_O_4_. The report shows that, except for pristine NiFe_2_O_4_, all the samples had a positive value of magnetocrystalline anisotropy, which increases monotonically as more Co is introduced, the material, and thus, may have a practical application as magnetic material [41]. Zhang et al. [42] considered the magnetic properties of La_x_NiCoFe_2−x_O_4_ (x = 0.025–0.125) synthesized via a sol-gel method, the substitution of La^3+^ effectively decreases the coercivity and increases the magnetization. On the other hand, Srinivasamurthy et al. [32] explored the phase and magnetic features of Ce-Sm doped Co-Ni nano spinel ferrite. They found that the magnetic measurements increased with increasing the dopant due to the improved superexchange interaction of A-B. Moreover, Hossain et al. [43] reported magnetic characteristics of Ni_0.7_Zn_0.2_Co_0.1_Fe_2−x_Gd_x_O_4_ (x = 0.00–0.12). Their findings showed that the saturation magnetization reduced while coercivity improved with adding Gd. Almessiere et al. implemented a research on magnetic merits of CoTm_x_Fe_2−x_O_4_ (0.0 ≤ x ≤ 0.08) and CoTb_x_Fe_2−x_O_4_ (x ≤ 0.10) NPs. Influence of synthesis method on structure and microstructural analysis was observed for nanosized substituted spinels [44]. The outcome of these studies demonstrated a strong influence of Tm and Tb individually on magnetic features of Co ferrites [45].

Although Tm and Tb are reported to influence the magnetic behaviors of ferrites solids [44,45], no clear trend of physicochemical properties due to substitution can be found thus far. Moreover, a bimetallic substitution into NiCoFe_2_O_4_ is scarcely explored. Herein, we introduced a rare earth Tb^3+^ and Tm^3+^ as (Co_0.5_Ni_0.5_)[Tm_x_Tb_x_Fe_2−2x_]O_4_ via ultrasonic technique. The material is characterized and studied the magnetic behavior as a function of the concentration of rare-earth dopants.

## 2. Materials and Methods

Ultrasound irradiation was employed to produce (Co_0.5_Ni_0.5_)[Tm_x_Tb_x_Fe_2−2x_]O_4_ (x = 0.00–0.05) NSFs. The Ni(NO_3_)_2_·6H_2_O, Co(NO_3_)_2_·6H_2_O, Tm_2_O_3,_ Tb_4_O_7,_ and Fe(NO_3_)_3_·9H_2_O are employed as sources of the metals. Initially, an appropriate amount of Tm_2_O_3_ and Tb_4_O_7_ were first dissolved in 10 mL acid solution containing concentrated HCl and HNO_3_, the admixture was carefully heated up at 200 °C through continued stirring for 1 h. Secondly, a required quantity of metal nitrates was thawed in 60 mL deionized (DI) H_2_O. Subsequently, the Tm_2_O_3_ and Tb_4_O_7_ solution was gradually adding to the nitrate solution, and the resultants solution stirred at room temperature. The pH was adjusted to 11 using an ammonia solution. The precursor mixture was undergoing ultrasound irradiation by ultrasonic homogenizer for 40 min and 20 kHz. The solid product was purified by washing using distilled water several times until a neutral pH is achieved. The solid is finally dried at 60 °C overnight to obtain NSFs [46,47].

The XRD Benchtop Rigaku Miniflex (Cu Kα line) was applied to identify the structure of the NSFs. The morphology and composition of the samples were analyzed using FEI Titan ST SEM and TEM equipped with an EDX spectrometer. Quantum Design PPMS DynaCool-9 coupled with VSM head was applied to study the magnetic properties of the products.

## 3. Results and Discussion

### 3.1. Structure

**Figure 1:**
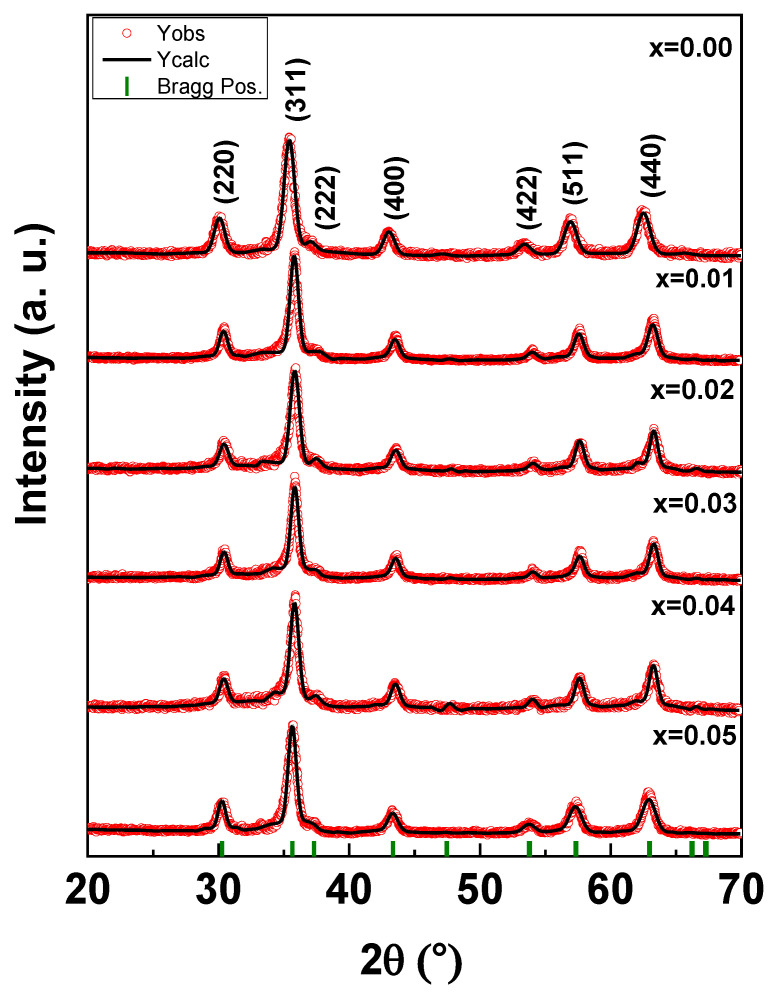
XRD powder patterns of (Co_0.5_Ni_0.5_)[Tm_x_Tb_x_Fe_2−2x_]O_4_ (x = 0.00–0.05) NSFs at room temperature.

Figure 1 depicts the XRD powder pattern of (Co_0.5_Ni_0.5_)[Tm_x_Tb_x_Fe_2−2x_]O_4_ (x = 0.00–0.05) NSFs. The characteristic peaks of the NSFs adequately matched with the JCPDS Card No 22-1086, and thus indicated the successful crystallization of the single phase of Co-Ni spinel ferrites. In addition, the Rietveld refinement revealed the absence of any trace rare earth oxides, suggesting the purity of the synthesized NSFs. It can be observed that, that the most intense peak at 2θ = 34.96° (x = 0.01–0.05) with a crystal plane (311) is shifted to a higher diffraction angle while x is increasing. Moreover, this peak becomes slightly broaden when increasing the ratio of the substituted ion; this is a characteristic of crystals with small particle size. Hence, the introduction of more Tm and Tb into the solid solution of the spinel ferrites hinders the crystal growth. A similar observation has been reported, where substitution using Gm^3+^, Sm^3+^ and Eu^3+^ resulted in a crystallite size reduction of Ni-Co ferrite [48]. On the other hand, cell parameters, cell volume, crystallite sizes, goodness fit (χ^2^), and R-factors have been evaluated via refinement through Match3 and a Full Proof program of X-ray experimental data as included in Table 1—lattice parameter (*a*), volume of unit cell (*V*), and average crystal size determined from XRD patterns (*D_XRD_*). A decrease in cell parameters and cell volume was observed when increasing the content of Tm^3+^ and Tb^3+^ (x = 0.0–0.05), this could be due to the deformation in spinel crystal by larger r_Tm3+_ (0.88 Å) and r_Tb3+_ (0.92 Å) in comparison with r_Fe3+_(0.64 Å) which caused depositary in the grain boundary and hinders the cell parameters growth [49]. The crystal sizes were computed with the Debye–Scherer formula by considering the maximum peak (311) and found in the range of 11–13 nm.

Bertaut method applied to investigate cation occupancy of Co_0.5_Ni_0.5_Tm_x_Tb_x_Fe_2−2x_O_4_ NSFs based on XRD data as following [50].

(1) $$\frac{I_{hkl}^{Obs.}}{I_{h’k’l’}^{Obs.}}\propto \frac{I_{hkl}^{Calc.}}{I_{h’k’l’}^{Calc.}}$$

where $I_{hkl}^{Obs.}$ and $I_{hkl}^{Calc.}$ are the practical and calculated intensities, respectively, for reflection (*hkl*). The ratio of XRD lines, *I*_220_/*I*_440_, and *I*_422_/*I*_400_ were used for calculating the cation distribution with the planes that sensitive to the cation distribution [50,51]. It is well-known that Spinel ferrites contain a crystallographic site, specifically Octahedral (B) and Tetrahedral (A). Table 1 listed the cation distribution of Co_0.5_Ni_0.5_Tm_x_Tb_x_Fe_2−2x_O_4_ system. The results revealed that for each value of x, the Fe^3+^, Ni^2+,^ and Co^2+^ ions occupied the A-site and B-site. Both Tm^3+^ and Tb^3+^ cations are found that they occupied B-site only due to their higher ionic radii [52].

### 3.2. Surface Morphology

**Figure 2:**
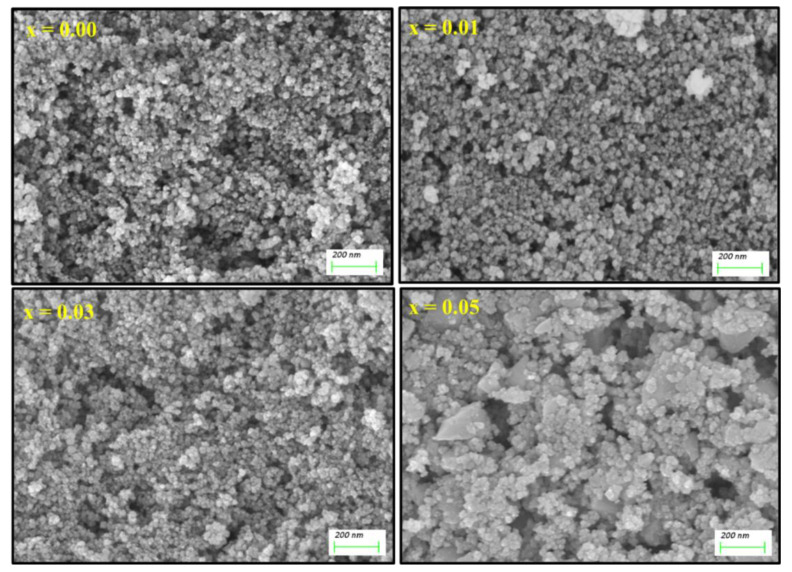
SEM images of (Co_0.5_Ni_0.5_)[Tm_x_Tb_x_Fe_2−2x_]O_4_ NSFs with x = 0.00, 0.01, 0.03 and 0.05.

**Figure 3:**
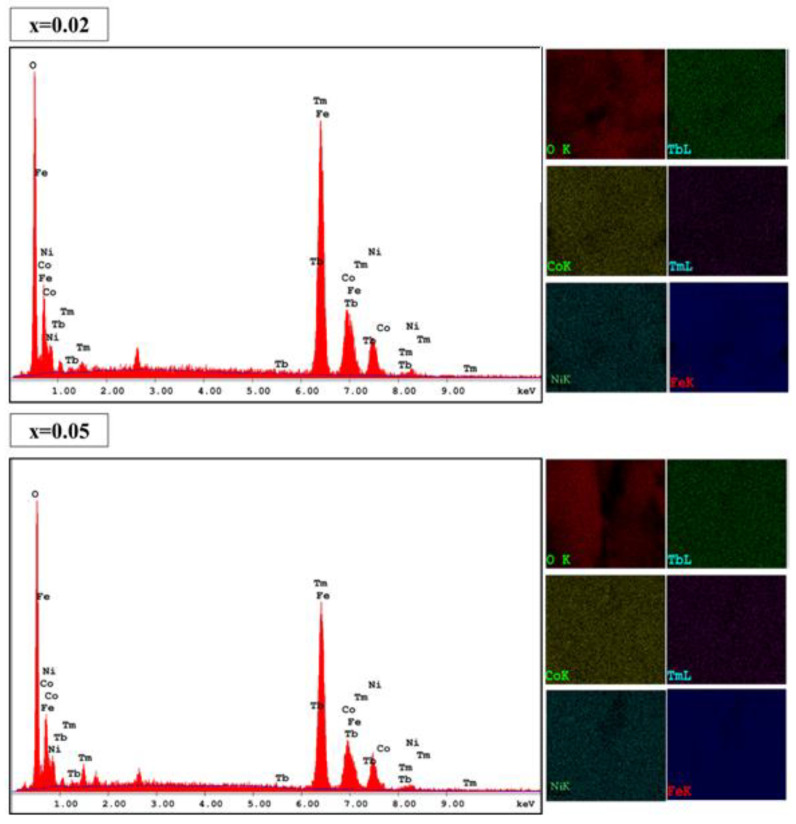
EDX spectra and elemental mappings of (Co_0.5_Ni_0.5_)[Tm_x_Tb_x_Fe_2−2x_]O_4_ NSFs with x = 0.02 and 0.05.

The morphological examination was conducted using SEM and the images for (Co_0.5_Ni_0.5_)[Tm_x_Tb_x_Fe_2−2x_]O_4_ (x = 0.00, 0.01, 0.03 and 0.05) NSFs were depicted in Figure 2. The crystals exhibited spherical shape morphology with an agglomerated appearance obviously due to ferrimagnetic character. Overall, the sample displayed an appreciable distribution of particle size, in the range 15 nm that agreed with the XRD analysis. Of note, the sample with the highest Tb^3+^ and Tm^3+^ doping (x = 0.5) contains some irregularly shaped nanocrystals, these crystals agglomerates and produce a secondary grain with a size range of 200–500 nm. The elemental weight percentage of (Co_0.5_Ni_0.5_)[Tm_x_Tb_x_Fe_2−2x_]O_4_ (x = 0.02 and 0.05) NSFs were evaluated using EDX elemental and mapping as shown in Figure 3. The EDX spectrum of all samples have verified the occurrence of Co, Ni, Tm, Tb, O and Fe elements in products. This also indicated the efficiency of the ultrasound synthesis method.

**Figure 4:**
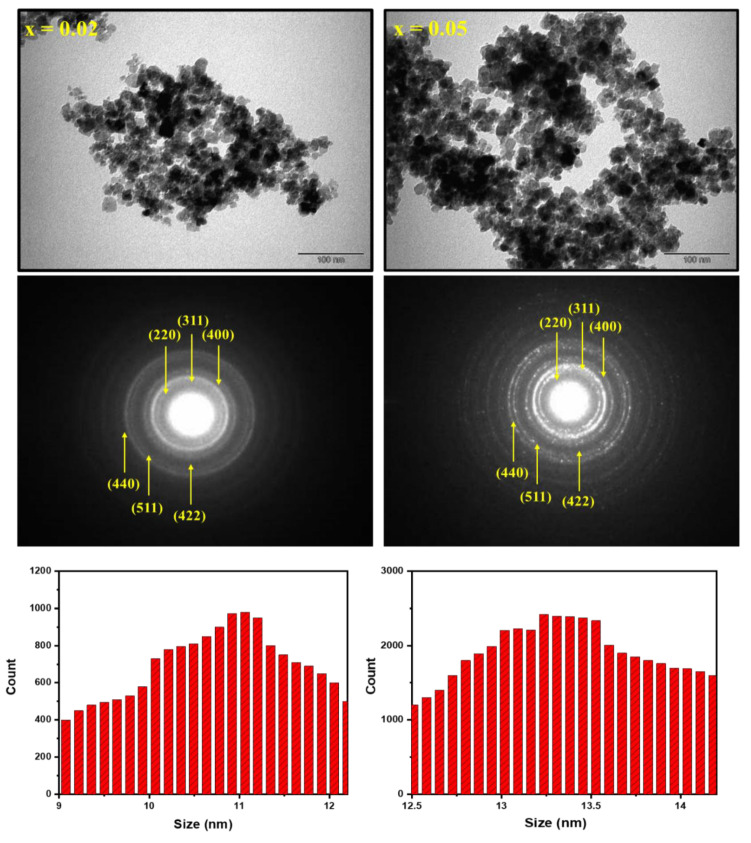
TEM, SAED, and size distribution histograms of (Co_0.5_Ni_0.5_)[Tm_x_Tb_x_Fe_2−2x_]O_4_ NSFs with x = 0.02 and 0.05.

To further study the structure, morphology, and particle size, TEM and SAED were employed on Co_0.5_Ni_0.5_)[Tm_x_Tb_x_Fe_2−2x_]O_4_ (x = 0.02 and 0.05) NSFs as seen in Figure 4. The TEM images disclosed the aggregation of cubic and spherical particles. Thus, observation of TEM images confirmed the uniformity of the particles. The particle size distribution histogram were calculated via Image J software and found that D_XRD_ of products have been within the range of 9–15 nm (Figure 4). The SAED showed a spotty bright ring with different intensities that confirmed the purity of the spinel NSFs ferrites structure. Accordingly, the index planes are consented with the XRD results.

### 3.3. Magnetic Properties

**Figure 5:**
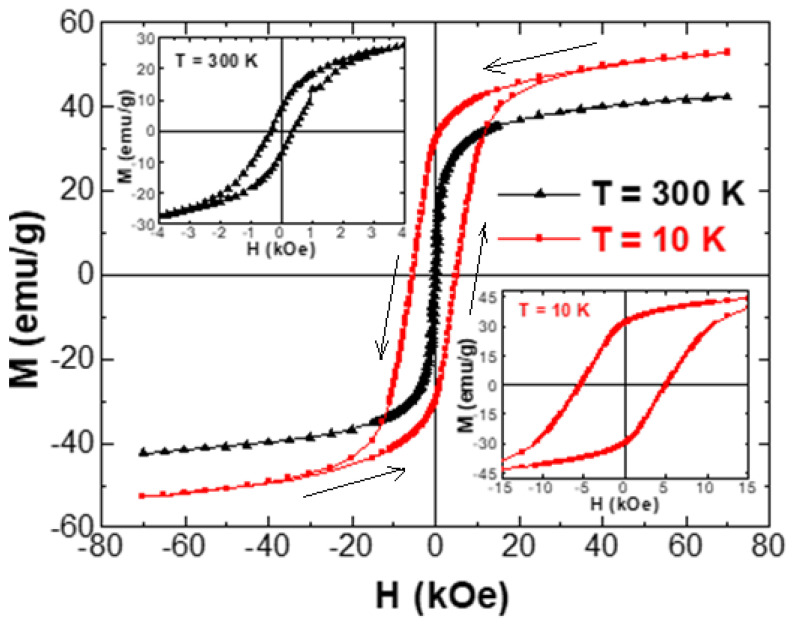
Magnetization versus applied magnetic field performed at T = 300 and 10 K for Co_0.5_Ni_0.5_Fe_2_O_4_ NSFs. The insets show magnified views of the region near the origin (H = 0 kOe).

**Figure 6:**
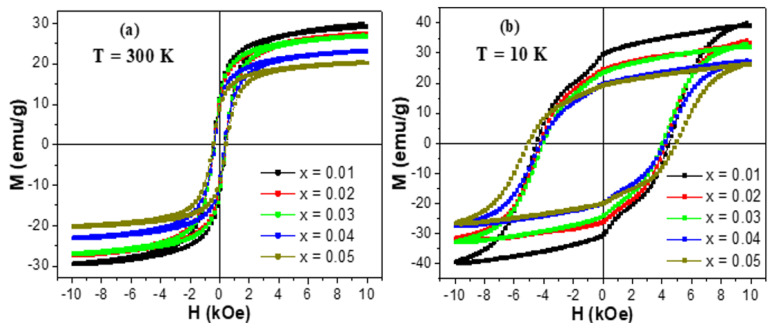
M-H hysteresis loops of (Co_0.5_Ni_0.5_)[Tm_x_Tb_x_Fe_2−x_]O_4_ NSFs with x = 0.01–0.05 performed at: (**a**) T = 300 K and (**b**) T = 10 K.

The experiments of magnetization versus the implemented magnetic field, M-H, were made at two selected temperatures (300 and 10 K). M-H hysteresis loops of unsubstituted and Tb/Tm-substituted Co-Ni spinel ferrites are existing in Figure 5 and Figure 6, respectively. The insets in Figure 5 show magnified regions near the origin (H = 0 kOe) so that the coercivity will be more visible. At both measurement temperatures, the different produced products displayed an opened M-H hysteresis loops with a clear and large coercivity, which reveal the ferrimagnetic (FM) behavior of various samples at 300 and 10 K. It is slightly obvious in various M-H hysteresis loops that the magnetization was not saturated easily even if the applied field is high. This observation is largely ascribed to the disordered or canted spins at the surface of NSFs, which are hard to be aligned along the direction of the field, which in turn will cause a non-saturated magnetization in the nanoparticles. In the following discussions, we referred to the magnetization at the maximum applied field as the saturation magnetization. Furthermore, Figure 6 showed a clear distortion in the region close to H = 0 kOe of M-H hysteresis loops performed at a lower temperature. Such distortion in M-H hysteresis loops is associated with a phenomenon of spins freezing, owing to the considerable time dependency of the remanent magnetization [53].

**Figure 7:**
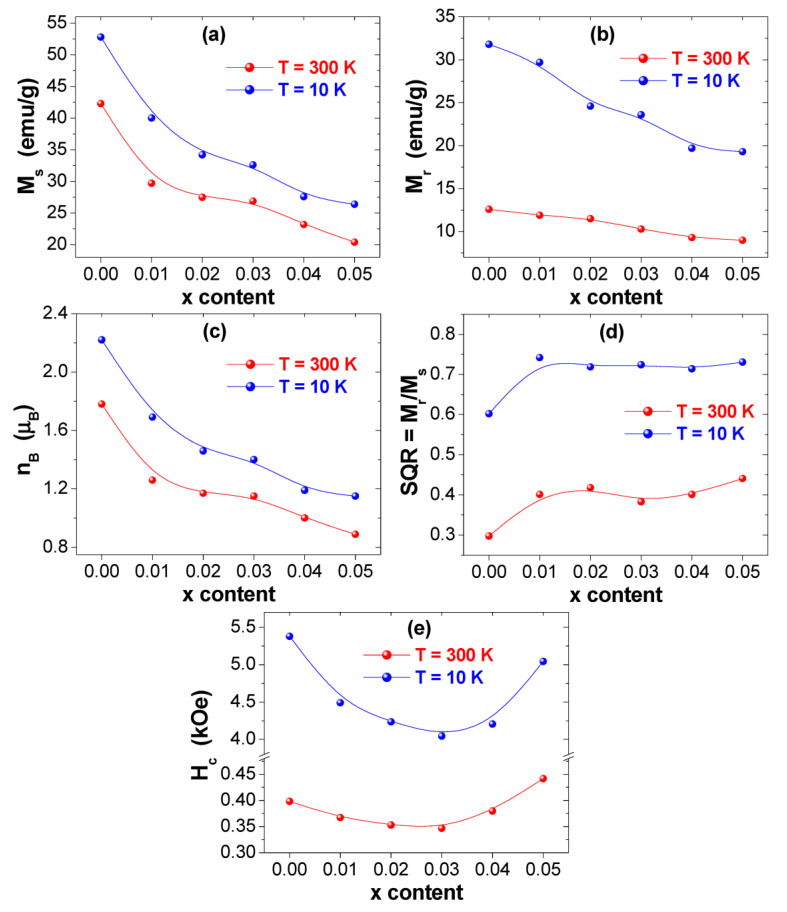
Variations of various deduced magnetic parameters of (Co_0.5_Ni_0.5_)[Tm_x_Tb_x_Fe_2−2x_]O_4_ with x = 0.00–0.05) NSFs at T = 300 and 10 K: (**a**) M_s_, (**b**) M_r_, (**c**) $n_{B}$, (**d**) SQR = M_r_/M_s_, (**e**) H_c_.

The several magnetic parameters are concluded from the performed M-H measurements and are offered in Figure 7. The variations of the saturation magnetization (M_s_) with respect to Tm-Tb amounts at 300 and 10 K are displayed in Figure 7a. It is clear that M_s_ value enhances with the reduction in the temperature of nanoparticles from 300 K down to 10 K. Generally, M_s_ below the Curie temperature (*T*_c_) obeys the Bloch’s law (Equation (2)) for bulky ferrimagnetic/ferromagnetic systems [54]:

(2) $$M_{s}\left(T\right)=M_{s}\left(0\right)\left[1-{\left(\frac{T}{T_{0}}\right)}^{\alpha }\right]$$

where $M_{s}\left(0\right)$ is the M_s_ value at T=0 K,$\;{\left(1/T_{0}\right)}^{\alpha }$ is the Bloch’s constant, α is the Bloch’s exponent, and $T_{0}$ is the temperature at which $M_{s}=0$. In line with this equation, when the temperature is decreasing, the magnetization will increase. Accordingly, the improvement in M_s_ values at lower temperatures compared to those at 300 K has essentially resulted from the reduction in the thermal fluctuations at lower temperatures [55,56]. Furthermore, the improvement in magnetization is accredited to the enhancement of spins order at the surface of Tb-Tm substituted Co_0.5_Ni_0.5_Fe_2_O_4_ nanoparticles [57]. Indeed, the spins moment of shells could additionally contribute to the resultant magnetization at lower temperatures, thus improving the total magnetization of NSFs [57].

The non-substituted sample (i.e., Co_0.5_Ni_0.5_Fe_2_O_4_) displayed M_s_ values of about ~42.3 and ~52.8 emu/g at T = 300 and 10 K, correspondingly. These registered values are lower than those attained in bulk Co_0.5_Ni_0.5_Fe_2_O_4_ system. These lower M_s_ values can be ascribed to the structural distortions in the surface such as dead surface layers and random spins canting in comparison to the bulk for which it can diminish the number of contributing moments [58,59,60]. Furthermore, the difference in the site occupancy of different cations in nanoparticulate and bulky systems is responsible for the dissimilarity in M_s_ values [61]. In fact, Co_0.5_Ni_0.5_Fe_2_O_4_ NPs do not exhibit an inverse spinel structure type like in bulk compounds, but they have a mixed spinel structure type. Consequently, this leads to the observed decrease in the M_s_ value. Nevertheless, the M_s_ values observed in our samples are larger than those found in Co-Ni spinel ferrite NSFs produced via sol-gel auto-combustion approach [62,63] and mechanochemical technique [64]. Almost near M_s_ values are observed in Co-Ni spinel ferrite NSFs generated through a one-pot modified-solvothermal approach [30].

M_s_ decreases at both 300 and 10 K as a function of Tm-Tb contents. M_s_ value decreases from ~42.3 to ~20.4 emu/g at 300 K and from ~52.8 to ~26.4 emu/g at 10 K. The minimum M_s_ values of ~20.4 and ~26.4 emu/g, respectively, at 300 and 10 K are belonging to the composition with x = 0.05. Generally, the magnetic properties of spinel ferrite nanoparticles are significantly influenced by the microstructure, the composition, the crystallites size, and the distribution of cations between the B- and A-sites in spinel structure [65,66,67]. Firstly, it was found that M_s_ decreases at both 300 and 10 K as a function of grains/crystallites size. The variation in M_s_ value could be also described based on the experimental values of the magneton number ($n_{B}$) per formula unit in Bohr magneton ($\mu_{B}$). The expression of $n_{B}$ is given as following [68]:

(3) $$n_{B}=\frac{Moleculor\;weight\;\times \;M_{s}}{5585}$$

The determined $n_{B}$ values are presented in Figure 7c. It is obvious that $n_{B}$, at both measurement temperatures 300 and 10 K, decreases with the rise in Tb^3+^ and Tm^3+^ contents, which reflect that the A-B superexchange interactions are weakened due to co-substitution effect. As evident from Figure 7a,c, the values of M_s_ and $n_{B}$ exposed similar tendencies as a function of Tb^3+^ and Tm^3+^ content.

The coercive fields (H_c_) deduced from M-H loops are presented in Figure 7e. One can remarkably observe an increase in the coercivity of all prepared nanoparticles with the diminution in the temperature from 300 down to 10 K, which indicates that the prepared nanoparticles become magnetically harder at very low temperatures. The H_c_ values for various synthesized products improve from 346.7–441.7 Oe at 300 K to 4044.4–5378.7 Oe at 10 K. The coercivity is a sensitive characteristic to the temperature of a product. In fact, additional magnetic moments are frozen within anisotropic directions at very low temperatures. Hence, the observed increment trend in H_c_ values is generally assigned to the effects of thermal fluctuations of the blocked moments across the anisotropy barriers. The effect of spins freezing is predominant at lower temperatures mainly below the blocking temperature (*T_B_*). This could develop because of the exchange coupling among the spins of the core and the surface. The coercive field (H_c_), for an assemblage of non-interacting three-dimensional single domain magnetic (SDM) NSFs and in a temperature interval lower than *T_B_*, can be expressed as follow [69,70]:

(4) $$H_{c}=H_{0}{\left(1-\frac{T}{T_{B}}\right)}^{1/2}$$

This expression is well-known as the Kneller’s law. It is a simple model form of thermal activation of the moments of nanoparticles across the anisotropy barriers. In Equation (4), the constant $H_{0}$ is the value of $H_{c}$ at T=0 K. It is evident from this equation that H_c_ value increases with the decrease in the temperature below *T_B_*. This indicates that the effects of thermal activation of the nanoparticle moments across the anisotropy barriers are prominent at lower temperatures. Nevertheless, one should also take into account that the anisotropy effect could have a great influence when one is investigating the nanoparticles at lower temperatures. Hence, other factors (in addition to the improvement of anisotropy), such as the intrinsic structural characteristics of NSFs comprising the volume distribution, the interactions between particles, and the random anisotropy, could also affect the thermal dependency of H_c_ in the case of NSFs [71].

In the current investigation, the variation of H_c_ values at both 300 and 10 K could be explained based on the cations redistribution at the octahedral and tetrahedral sites and on the change of grains/crystallites size [47,65]. The non-substituted Co_0.5_Ni_0.5_Fe_2_O_4_ NSFs displayed H_c_ values of ~398.2 and ~5378.7 Oe at 300 and 10 K, respectively. Initially, H_c_ diminishes with the increase of Tb-Tm content up to x = 0.03. Afterward, it increases with further increasing Tb-Tm substituting contents. Largely, the variation in coercivity for nanoparticles is proportional to the crystallites/grains size [72]. At Tb-Tm substitution content lower than 0.3, it is noticed that H_c_ decreases with the decrease in grains/crystallites size as found in XRD and SEM investigations. However, for x ≥ 0.3, the coercivity and grains/crystallites size does not follow each other. The redistribution of cations at A and B- sites greatly influences the H_c_ of the Co-Ni ferrite NSFs. Accordingly, the improvement of H_c_ at higher Tb-Tm content (i.e., x ≥ 0.3) could be attributed to the existence of Co^2+^ and Ni^2+^ cations at the B-sites that lead to great values of magnetic anisotropy and coercivity because of the strong L–S coupling of bivalent^+^ cations at B-site. According to the observed results, the synthesized (Co_0.5_Ni_0.5_)[Tm_x_Tb_x_Fe_2−x_]O_4_ NSFs could be considered as promising candidates to be used for room temperature magnetic applications and magnetic recording media [73].

The variations of the M_r_ value and the squareness ratio (SQR = M_r_/M_s_) against Tm-Tb contents are displayed in Figure 7b,d. It is observed that both M_r_ and SQR values for different samples increase with reducing the temperature from 300 down to 10 K, which indicates that the strength of the magnetic anisotropy intensifies at lower temperatures [74]. The M_r_ values are around ~12.6 and ~31.8 emu/g, respectively, at 300 and 10 K for un-substituted sample (x = 0.00). As observed in Figure 7a,b, the variations in M_r_ and M_s_ values exposed similar tendency versus Tb^3+^ and Tm^3+^ contents. Indeed, M_r_ values are maximum for the non-substituted Co_0.5_Ni_0.5_Fe_2_O_4_ NPs and then decreased upon Tb-Tm co-substitution. As shown via the morphological observations, the densities of grains of the diverse prepared products are very close. Therefore, the decline in M_r_ values is largely dependent on the evolution of M_s_ and the net alignment of grains magnetization induced by weak exchange interactions between particles. According to the Stoner–Wohlfarth concept for non-interacting particles with randomly oriented easy axes, SQR is around ~0.8 for cubic anisotropy and ~0.5 for uniaxial anisotropy [74]. Furthermore, a SQR value lower than 0.5 reflects that a multi-magnetic domain (MMD) structure is formed in which the motion of domain walls permits for an easier change in the orientation with the applied field, whereas, SQR value ≥0.5 implies that the system is around the single magnetic domain (SMD) size [75,76]. As shown in Figure 7d, SQR values for the synthesized products are in the interval of 0.298–0.441 (i.e., lower than 0.5) at T = 300 K, indicating that these products display a MMD structure with uniaxial anisotropy. However, SQR values are exceeding the value of 0.5 at 10 K but are still lower than 0.8, which implies that these products have a virtually SMD structure with the coexistence of cubic and uniaxial anisotropy.

## 4. Conclusions

The interdependence of Tm-Tb on the phase and magnetic features of Co-Ni NSFs as a new study has been presented. (Co_0.5_Ni_0.5_)[Tm_x_Tb_x_Fe_2−2x_]O_4_ (x = 0.00–0.05) NSFs. NSFs were produced via ultrasound irradiation procedure. X-ray diffraction presented a single-phase formation of NSFs. It was found that the cell parameters decrease with the rise in the content of substitution ions. The morphology and grain size were estimated by SEM and TEM. The M-H hysteresis loops of various prepared nanoparticles showed a ferrimagnetic behavior at both measurement temperatures 300 and 10K. It was found that M_s_, M_r_, $n_{B}$ decreased as a function of Tb-Tm content. H_c_ values decreased with the increase of *x* content up to 0.03 and then increased with the further rise of Tb-Tm content. The variations in these magnetic parameters are successfully described based on the redistribution of cations, variations crystallites and/or grains size, canting effects, surface spins effects, super-exchange interaction strength, etc. The obtained magnetic results are found to be encouraging for the possible use of these products in room temperature magnetic applications and magnetic recording media.

## Figures and Tables

**Table 1:** Refined structural parameters and cation distribution calculations for (Co_0.5_Ni_0.5_)[Tm_x_Tb_x_Fe_2__−2x_]O_4_ (x = 0.00–0.05) NSFs.

x	*a* (Å)	*V* (Å)^3^	*D_XRD_* (nm) ±0.05	*χ* ^2^ *(chi* ^2^ *)*	*R_Bragg_*	Cations Distribution	
						A-Site	B-Site
0.00	8.357 (3)	583.73	23.9	1.21	12.4	Co_0.1_Ni_0.1_Fe_0.8_	Co_0.4_Ni_0.4_Fe_1.2_
0.01	8.336 (4)	579.34	13.32	1.85	1.74	Co_0.05_Ni_0.15_Fe_0.8_	Co_0.45_Ni_0.35_Tm_0.01_Tb_0.01_Fe_1.18_
0.02	8.320 (3)	575.99	11.24	1.35	4.12	Co_0.05_Ni_0.15_Fe_0.8_	Co_0.45_Ni_0.35_Tm_0.02_Tb_0.02_Fe_1.16_
0.03	8.316 (5)	575.21	13.88	1.05	0.89	Co_0.05_Ni_0.15_Fe_0.8_	Co_0.45_Ni_0.35_Tm_0.03_Tb_0.03_Fe_1.14_
0.04	8.316 (1)	575.12	12.48	1.08	2.20	Co_0.05_Ni_0.15_Fe_0.8_	Co_0.45_Ni_0.35_Tm_0.04_Tb_0.04_Fe_1.12_
0.05	8.310 (6)	573.97	13.93	1.04	3.48	Co_0.05_Ni_0.15_Fe_0.8_	Co_0.45_Ni_0.35_Tm_0.05_Tb_0.05_Fe_1.10_

## References

[1] Guo H.-H., Zhou D., Du C., Wang P.-J., Liu W.-F., Pang L.-X., Wang Q.-P., Su J.-Z., Singh C., Trukhanov S. (2020). Temperature stable Li_2_Ti_0.75_(Mg_1/3_Nb_2/3_)_0.25_O_3_-based microwave dielectric ceramics with low sintering temperature and ultra-low dielectric loss for dielectric resonator antenna applications. J. Mater. Chem. C.

[2] Dukenbayev K., Korolkov I.V., Tishkevich D.I., Kozlovskiy A.L., Trukhanov S.V., Gorin Y.G., Shumskaya E.E., Kaniukov E.Y., Vinnik D.A., Zdorovets M.V. (2019). Fe_3_O_4_ Nanoparticles for Complex Targeted Delivery and Boron Neutron Capture Therapy. Nanomaterials.

[3] Salem M.M., Panina L.V., Trukhanova E.L., Darwish M.A., Morchenko A.T., Zubar T.I., Trukhanov S.V., Trukhanov A.V. (2019). Structural, electric and magnetic properties of (BaFe_11.9_Al_0.1_O_19_)_1-x_-(BaTiO_3_)_x_ composites. Comp. Part B Eng..

[4] Ketsko V.A., Beresnev E.N., Kop’eva M.A., Rjabkova L.V., Baranchicov A.E., Stognij A.I., Trukhanov A.V., Kuznetsov N.T. (2010). Specifics of the pyrohydrolytic and solid-phase syntheses of solid solutions in the (MgGa_2_O_4_)x(MgFe_2_O_4_)_1-x_ system. Rus. J. Inorg. Chem..

[5] Nipan G.D., Ketsko V.A., Stognij A.I., Trukhanov A.V., Kol’tsova T.N., Kop’eva M.A., Elesina L.V., Kuznetsov T.N. (2010). Properties of Mg(Fe_1-X_Ga_X_)_2_O_4+__δ_ Solid solutions in stable and metastable state. Inorg. Mater..

[6] Gang C., Zheng H., Zhao H., Ni Y., Pocs C.A., Zhang Y., Ye F., Hoffmann C., Wang X., Lee M. (2020). Quantum liquid from strange frustration in the trimer magnet Ba_4_Ir_3_O_10_. NPJ Quantum Mater..

[7] Karpinsky D.V., Silibin M.V., Trukhanov S.V., Trukhanov A.V., Zhaludkevich A.L., Latushka S.I., Zhaludkevich D.V., Khomchenko V.A., Alikin D.O., Abramov A.S. (2020). Peculiarities of the crystal structure evolution of BiFeO_3_-BaTiO_3_ ceramics across structural phase transitions. Nanomaterials.

[8] Pisarev R.V., Moskvin A.S., Kalashnikova A.M., Rasing T. (2009). Charge transfer transitions in multiferroic BiFeO_3_ and related ferrite insulators. Phys. Rev. B.

[9] Fodouop F.K., Fouokeng G.C., Ateuafack M.E., Tchoffo M., Fai L.C. (2020). Metamagnetoelectric effect in multiferroics A_2_Cu_2_Mo_3_O_12_ (A=Rb and Cs) quantum spin chain. Phys. B.

[10] Da Silveira Lacerda L.H., de Lazaro S.R. (2020). Magneto-optical coupling and Kerr effect in PbNiO_3_, PbCrO_3_, and PbMnO_3_ multiferroics: An excited-states approach. J. Magn. Magn. Mater..

[11] Trukhanov S.V., Trukhanov A.V., Kostishin V.G., Panina L.V., Kazakevich I.S., Turchenko V.A., Kochervinskiy V.V. (2016). Coexistence of spontaneous polarization and magnetization in substituted M-type hexaferrites BaFe_12–x_Al_x_O_19_ (x ≤ 1.2) at room temperature. JETP Lett..

[12] Turchenko V., Trukhanov A., Trukhanov S., Balasoiu M., Lupu N. (2019). Correlation of crystalline and magnetic structures of barium ferrites with dual ferroic properties. J. Magn. Magn. Mater..

[13] Turchenko V., Kostishyn V.G., Trukhanov S., Damay F., Porcher F., Balasoiu M., Lupu N., Bozzo B., Fina I., Trukhanov A. (2020). Crystal and magnetic structures, magnetic and ferroelectric properties of strontium ferrite partially substituted with in ions. J. Alloys Compd..

[14] Yakovenko O.S., Matzui L.Y., Vovchenko L.L., Lozitsky O.V., Prokopov O.I., Lazarenko O.A., Zhuravkov A.V., Oliynyk V.V., Launets V.L., Trukhanov S.V. (2018). Electrophysical properties of epoxy-based composites with graphite nanoplatelets and magnetically aligned magnetite. Mol. Cryst. Liq. Cryst..

[15] Darwish M.A., Trukhanov A.V., Senatov O.S., Morchenko A.T., Saafan S.A., Astapovich K.A., Trukhanov S.V., Trukhanova E.L., Pilyushkin A.A., Sombra A.S.B. (2020). Investigation of AC-measurements of epoxy/ferrite composites. Nanomaterials.

[16] Trukhanov A.V., Astapovich K.A., Turchenko V.A., Almessiere M.A., Slimani Y., Baykal A., Sombra A.S.B., Zhou D., Jotania R.B., Singh C. (2020). Influence of the dysprosium ions on structure, magnetic characteristics and origin of the reflection losses in the Ni-Co spinels. J. Alloys Compd..

[17] Gao F. (2019). An Overview of surface-functionalized magnetic nanoparticles: Preparation and application for wastewater treatment. ChemistrySelect.

[18] López-Ortega A., Lottini E., Fernandez C.d.J., Sangregorio C. (2015). Exploring the magnetic properties of cobalt-ferrite nanoparticles for the development of a rare-earth-free permanent magnet. Chem. Mater..

[19] Dong B., Li M., Xiao C., Ding D., Gao G., Ding S. (2016). Tunable growth of perpendicular cobalt ferrite nanosheets on reduced graphene oxide for energy storage. Nanotechnology.

[20] Arbab A.S., Jordan E.K., Wilson L.B., Yocum G.T., Lewis B.K., Frank J.A. (2004). In Vivo trafficking and targeted delivery of magnetically labeled stem cells. Hum. Gene Ther..

[21] Gloag L., Mehdipour M., Chen D., Tilley R.D., Gooding J.J. (2019). Advances in the application of magnetic nanoparticles for sensing. Adv. Mater..

[22] Mahdi T.S., Kadhim F.J. (2020). Effect depositions parameters on the characteristics of Ni_0.5_Co_0.5_Fe_2_O_4_ nanocomposite films prepared by DC reactive magnetron Co-sputtering technique. Iraqi J. Phys..

[23] Harris V.G. (2011). Modern microwave ferrites. IEEE Trans. Magn..

[24] Tong G., Liu Y., Cui T., Li Y., Zhao Y., Guan J. (2016). Tunable dielectric properties and excellent microwave absorbing properties of elliptical Fe_3_O_4_ nanorings. Appl. Phys. Lett..

[25] Datt G., Kotabage C., Abhyankar A. (2017). Ferromagnetic resonance of NiCoFe_2_O_4_ nanoparticles and microwave absorption properties of flexible NiCoFe_2_O_4_–carbon black/poly(vinyl alcohol) composites. Phys. Chem. Chem. Phys..

[26] Osaka T., Takai M., Hayashi K., Ohashi K., Saito M., Yamada K. (1998). A soft magnetic CoNiFe film with high saturation magnetic flux density and low coercivity. Nature.

[27] Mozaffari M., Amighian J., Darsheshdar E. (2014). Magnetic and structural studies of nickel-substituted cobalt ferrite nanoparticles, synthesized by the sol-gel method. J. Magn. Magn. Mater..

[28] Almeida T.P., Fay M.W., Zhu Y., Brown P.D. (2012). Hydrothermal synthesis of mixed cobalt-nickel ferrite nanoparticles. J. Phys. Conf. Ser..

[29] Muscas G., Yaacoub N., Concas G., Sayed F., Hassan R.S., Greneche J.-M., Cannas C., Musinu A., Foglietti V., Casciardi S. (2015). Evolution of the magnetic structure with chemical composition in spinel iron oxide nanoparticles. Nanoscale.

[30] Datt G., Bishwas M.S., Raja M.M., Abhyankar A. (2016). Observation of magnetic anomalies in one-step solvothermally synthesized nickel–cobalt ferrite nanoparticles. Nanoscale.

[31] Bahgat M., Paek M.-K., Park C.-H., Pak J.-J. (2008). Thermal synthesis of nanocrystalline (Co_x_Ni_1-x_)_y_Fe_1-y_ KOVAR alloy through gaseous reduction of mixed oxides. Mater. Trans..

[32] Srinivasamurthy K., Angadi V.J., Kubrin S., Matteppanavar S., Kumar P.M., Rudraswamy B. (2018). Evidence of enhanced ferromagnetic nature and hyperfine interaction studies of Ce-Sm doped Co-Ni ferrite nanoparticles for microphone applications. Ceram. Int..

[33] Asiri S., Sertkol M., Guner S., Gungunes H., Batoo K., Saleh T.A., Sozeri H., Almessiere M.A., Manikandan A., Baykal A. (2018). Hydrothermal synthesis of Co_y_Zn_y_Mn_1-2y_Fe_2_O_4_ nanoferrites: Magneto-optical investigation. Ceram. Int..

[34] Auwal I., Erdemi H., Sözeri H., Güngüneş H., Baykal A. (2016). Magnetic and dielectric properties of Bi^3+^ substituted SrFe_12_O_19_ hexaferrite. J. Magn. Magn. Mater..

[35] Maity D., Ding J., Xue J.-M. (2008). Synthesis of magnetite nanoparticles by thermal decomposition: Time, temperature, surfactant and solvent effects. Func. Mater. Lett..

[36] Biehl P., Von der Lühe M., Dutz S., Schacher F.H. (2018). Synthesis, characterization, and applications of magnetic nanoparticles featuring polyzwitterionic coatings. Polymers.

[37] Wang Y., Nkurikiyimfura I., Pan Z. (2015). Sonochemical synthesis of magnetic nanoparticles. Chem. Eng. Comm..

[38] Faraji M., Yamini Y., Rezaee M. (2010). Magnetic nanoparticles: Synthesis, stabilization, functionalization, characterization, and applications. J. Iran. Chem. Soc..

[39] Nkurikiyimfura I., Wang Y., Pan Z. (2013). Effect of chain-like magnetite nanoparticle aggregates on thermal conductivity of magnetic nanofluid in magnetic field. Exp. Therm. Fluid Sci..

[40] Marchegiani G., Imperatori P., Mari A., Pilloni L., Chiolerio A., Allia P., Tiberto P., Suber L. (2012). Sonochemical synthesis of versatile hydrophilic magnetite nanoparticles. Ultrason. Sonochem..

[41] De Biasi R.S., de Souza Lopes R.D. (2016). Magnetocrystalline anisotropy of NiCoFe_2_O_4_ nanoparticles. Ceram. Int..

[42] Zhang W., Sun A., Zhao X., Suo N., Yu L., Zuo Z. (2019). Structural and magnetic properties of La^3+^ ion doped Ni-Cu-Co nano ferrites prepared by sol-gel auto-combustion method. J. Sol Gel Sci. Technol..

[43] Hossain M., Khan M., Nahar A., Ali M., Matin M., Hoque S., Hakim M., Jamil A. (2020). Tailoring the properties of Ni-Zn-Co ferrites by Gd^3+^ substitution. J. Magn. Magn. Mater..

[44] Almessiere M.A., Trukhanov A.V., Khan F.A., Slimani Y., Tashkandi N., Turchenko V.A., Zubar T.I., Tishkevich D.I., Trukhanov S.V., Panina L.V. (2020). Correlation between microstructure parameters and anti-cancer activity of the [Mn_0.5_Zn_0.5_](Eu_x_Nd_x_Fe_2-2x_)O_4_ nanoferrites produced by modified sol-gel and ultrasonic methods. Ceram. Int..

[45] Sadaqat A., Almessiere M., Slimani Y., Guner S., Sertkol M., Albetran H., Baykal A., Shirsath S.E., Ozcelik B., Ercan I. (2019). Structural, optical and magnetic properties of Tb^3+^ substituted Co nanoferrites prepared via sonochemical approach. Ceram. Int..

[46] Almessiere M., Slimani Y., Guner S., Sertkol M., Korkmaz A.D., Shirsath S.E., Baykal A. (2019). Sonochemical synthesis and physical properties of Co_0.3_Ni_0.5_Mn_0.2_Eu_x_Fe_2−x_O_4_ nano-spinel ferrites. Ultrason. Sonochem..

[47] Almessiere M., Slimani Y., Kurtan U., Guner S., Sertkol M., Shirsath S.E., Akhtar S., Baykal A., Ercan I. (2019). Structural, magnetic, optical properties and cation distribution of nanosized Co_0.7_Zn_0.3_Tm_x_Fe_2−x_O_4_ (0.0≤x≤0.04) spinel ferrites synthesized by ultrasonic irradiation. Ultrason. Sonochem..

[48] Tanbir K., Ghosh M.P., Singh R.K., Kar M., Mukherjee S. (2020). Effect of doping different rare earth ions on microstructural, optical, and magnetic properties of nickel-cobalt ferrite nanoparticles. J. Mater. Sci. Mater. Electron..

[49] Kadam A., Mande V.K., Kadam S., Kadam R., Shirsath S.E., Borade R.B. (2020). Influence of gadolinium (Gd^3+^) ion substitution on structural, magnetic and electrical properties of cobalt ferrites. J. Alloys Compd..

[50] Shirsath S.E., Mane M., Yasukawa Y., Liu X., Morisako A. (2013). Chemical tuning of structure formation and combustion process in CoD_y0.1_Fe_1.9_O_4_ nanoparticles: Influence@pH. J. Nanoparticle Res..

[51] Shirsath S.E., Mane M.L., Yasukawa Y., Liu X., Morisako A. (2014). Self-ignited high temperature synthesis and enhanced super-exchange interactions of Ho^3+^–Mn^2+^–Fe^3+^–O^2−^ ferromagnetic nanoparticles. Phys. Chem. Chem. Phys..

[52] Klein L., Aparicio M., Jitianu A. (2018). Handbook of Sol-Gel Science and Technology.

[53] Abdallah H., Msomi J., Moyo T., Dolo J., Lančok A. (2011). Mössbauer and magnetic studies of Mn_0.1_Sr_0.2_Co_0.7_Fe_2_O_4_ nanoferrite. Hyperfine Interact..

[54] Bloch F. (1930). Zur Theorie des Ferromagnetismus. Zeitschrift für Physik.

[55] Trukhanov A.V., Algarou N.A., Slimani Y., Almessiere M.A., Baykal A., Tishkevich D.I., Vinnik D.A., Vakhitov M.G., Klygach D.S., Silibin M.V. (2020). Peculiarities of the microwave properties of hard-soft functional composites SrTb_0.01_Tm_0.01_Fe_11.98_O_19_-AFe_2_O_4_ (A = Co, Ni, Zn, Cu and Mn). RSC Adv..

[56] Almessiere M.A., Slimani Y., Gungunes H., Manikandan A., Baykal A. (2019). Investigation of the effects of Tm^3+^ on the structural, microstructural, optical, and magnetic properties of Sr hexaferrites. Res. Phys..

[57] Tung L., Kolesnichenko V., Caruntu D., Chou N., O’Connor C., Spinu L. (2003). Magnetic properties of ultrafine cobalt ferrite particles. J. Appl. Phys..

[58] Franco A., e Silva F. (2010). High temperature magnetic properties of cobalt ferrite nanoparticles. Appl. Phys. Lett..

[59] Coey J. (1987). Noncollinear spin structures. Can. J. Phys..

[60] Slimani Y., Almessiere M., Nawaz M., Baykal A., Akhtar S., Ercan I., Belenli I. (2019). Effect of bimetallic (Ca, Mg) substitution on magneto-optical properties of NiFe_2_O_4_ nanoparticles. Ceram. Int..

[61] Nakagomi F., Da Silva S., Garg V., Oliveira A., Morais P., Franco A., Lima E. (2007). The influence of cobalt population on the structural properties of Co_x_Fe_3−x_O_4_. J. Appl. Phys..

[62] Saffari F., Kameli P., Rahimi M., Ahmadvand H., Salamati H. (2015). Effects of Co-substitution on the structural and magnetic properties of NiCo_x_Fe_2−x_O_4_ ferrite nanoparticles. Ceram. Int..

[63] Algarou N.A., Slimani Y., Almessiere M.A., Sadaqat A., Trukhanov A.V., Gondal M.A., Hakeem A.S., Trukhanov S.V., Vakhitov M.G., Klygach D.S. (2020). Functional Sr_0.5_Ba_0.5_Sm_0.02_Fe_11.98_O_4_/x(Ni_0.8_Zn_0.2_Fe_2_O_4_) hard-soft ferrite nanocomposites: Structure, magnetic and microwave properties. Nanomaterials.

[64] Chermahini M.D., Baghbaderani H.A., Shahraki M.M., Kazazi M. (2019). Low temperature sintering of magnetic Ni_0.5_Co_0.5_Fe_2_O_4_ ceramics prepared from mechanochemically synthesized nanopowders. Ceram. Int..

[65] Kumar Y., Shirage P.M. (2017). Highest coercivity and considerable saturation magnetization of CoFe_2_O_4_ nanoparticles with tunable band gap prepared by thermal decomposition approach. J. Mater. Sci..

[66] Almessiere M., Slimani Y., Güner S., Baykal A., Ercan I. (2019). Effect of dysprosium substitution on magnetic and structural properties of NiFe_2_O_4_ nanoparticles. J. Rare Earths.

[67] Slimani Y., Almessiere M., Güner S., Tashkandi N., Baykal A., Sarac M., Nawaz M., Ercan I. (2019). Calcination effect on the magneto-optical properties of vanadium substituted NiFe_2_O_4_ nanoferrites. J. Mater. Sci. Mater. Electron..

[68] Slimani Y., Unal B., Almessiere M., Korkmaz A.D., Shirsath S.E., Yasin G., Trukhanov A., Baykal A. (2020). Investigation of structural and physical properties of Eu^3+^ ions substituted Ni_0.4_Cu_0.2_Zn_0.4_Fe_2_O_4_ spinel ferrite nanoparticles prepared via sonochemical approach. Res. Phys..

[69] Maaz K., Mumtaz A., Hasanain S., Bertino M. (2010). Temperature dependent coercivity and magnetization of nickel ferrite nanoparticles. J. Magn. Magn. Mater..

[70] Slimani Y., Almessiere M., Güner S., Kurtan U., Shirsath S.E., Baykal A., Ercan I. (2020). Magnetic and microstructural features of Dy^3+^ substituted NiFe_2_O_4_ nanoparticles derived by sol-gel approach. J. Sol Gel Sci. Technol..

[71] Iglesias O., Labarta A., Batlle X. (2008). Exchange bias phenomenology and models of core/shell nanoparticles. J. Nanosci. Nanotechnol..

[72] Slimani Y., Almessiere M., Korkmaz A.D., Guner S., Güngüneş H., Sertkol M., Manikandan A., Yildiz A., Akhtar S., Shirsath S.E. (2019). Ni_0.4_Cu_0.2_Zn_0.4_Tb_x_Fe_2-x_O_4_ nanospinel ferrites: Ultrasonic synthesis and physical properties. Ultrason. Sonochem..

[73] Tegus O., Brück E., Buschow K., De Boer F. (2002). Transition-metal-based magnetic refrigerants for room-temperature applications. Nature.

[74] Osman N.S., Moyo T. (2016). Temperature dependence of coercivity and magnetization of Sr_1/3_Mn_1/3_Co_1/3_Fe_2_O_4_ ferrite nanoparticles. J. Supercond. Nov. Magn..

[75] Chauhan C.C., Kagdi A.R., Jotania R.B., Upadhyay A., Sandhu C.S., Shirsath S.E., Meena S.S. (2018). Structural, magnetic and dielectric properties of Co-Zr substituted M-type calcium hexagonal ferrite nanoparticles in the presence of α-Fe_2_O_3_ phase. Ceram. Int..

[76] Almessiere M., Slimani Y., Korkmaz A., Taskhandi N., Sertkol M., Baykal A., Shirsath S.E., Ercan İ., Ozcelik B. (2019). Sonochemical synthesis of Eu^3+^ substituted CoFe_2_O_4_ nanoparticles and their structural, optical and magnetic properties. Ultrason. Sonochem..

